# The biogeography of soil archaeal communities on the eastern Tibetan Plateau

**DOI:** 10.1038/srep38893

**Published:** 2016-12-13

**Authors:** Yu Shi, Jonathan M. Adams, Yingying Ni, Teng Yang, Xin Jing, Litong Chen, Jin-Sheng He, Haiyan Chu

**Affiliations:** 1 State Key Laboratory of Soil and Sustainable Agriculture, Institute of Soil Science, Chinese Academy of Sciences, East Beijing Road 71, Nanjing 210008, China; 2 Department of Biological Sciences, Seoul National University, Gwanak, Seoul 151, Republic of Korea; 3 Department of Ecology, College of Urban and Environmental Sciences and Key Laboratory for Earth Surface Processes of the Ministry of Education, Peking University, 5 Yiheyuan Road, Beijing 100871, China; 4 Key Laboratory of Adaptation and Evolution of Plateau Biota, Northwest Institute of Plateau Biology, Chinese Academy of Sciences, 23 Xinning Road, Xining 810008, China

## Abstract

The biogeographical distribution of soil bacterial communities has been widely investigated. However, there has been little study of the biogeography of soil archaeal communities on a regional scale. Here, using high-throughput sequencing, we characterized the archaeal communities of 94 soil samples across the eastern Tibetan Plateau. Thaumarchaeota was the predominant archael phylum in all the soils, and Halobacteria was dominant only in dry soils. Archaeal community composition was significantly correlated with soil moisture content and C:N ratio, and archaeal phylotype richness was negatively correlated with soil moisture content (r = −0.47, P < 0.01). Spatial distance, a potential measure of the legacy effect of evolutionary and dispersal factors, was less important than measured environmental factors in determining the broad scale archaeal community pattern. These results indicate that soil moisture and C:N ratio are the key factors structuring soil archaeal communities on the eastern Tibetan Plateau. Our findings suggest that archaeal communities have adjusted their distributions rapidly enough to reach range equilibrium in relation to past environmental changes e.g. in water availability and soil nutrient status. This responsiveness may allow better prediction of future responses of soil archaea to environmental change in these sensitive ecosystems.

Studying the distribution of soil microbial communities across the space and time may give important indications of the processes that dominate microbial ecology^1^. Various studies have been conducted to compare patterns in microbial distributions to those commonly observed for animal and plant taxa^2^,^3^. These have included studies of soil microbial communities across North America^4^, the Arctic^5^, Britain^6^ and the western Tibetan Plateau^7^. So far, these seem mostly to have demonstrated the principle that “everything is everywhere, but the environment selects^8^”8. In other words, dispersal does not emerge as an important limiting factor, and microbial community composition is strongly influenced by contemporary site-specific environmental conditions^4^,^5^,^9^,^10^. However, such work has focused mainly on bacterial distribution patterns. Much less is known about how archaeal communities are distributed on a broad scale.

Archaea, one of the three domains of life, were once thought to be confined to extreme environments, but are now known to occur in marine^11^, seawater^12^,^13^, lake sediments^14^, and soils^15^. In an early study on archaeal distributions, a high-resolution multi locus sequence analysis revealed that, on a global scale, populations of hyperthermophilic microorganisms were isolated from one another by geographic barriers in soils^16^. When 105 soil samples were collected from 2 habitat types (non-flooded soil and flooded soil) in China, it was found that longitude was an important factor predicting the archaeal distribution in these two habitats^17^. The authors suggested that archaeal community composition was more influenced by dispersal limitation between these very isolated locations, rather than variation in environment. A more recent study of archaeal distributions along a steep precipitation gradient, ranging from the Negev Desert in the south of Israel to the Mediterranean forests in the north, suggested that archaeal community composition was mostly determined by environment, being particularly strongly correlated with soil carbon content and the vegetation cover^18^. A very broad scale investigation of the global distribution of soil archaea strongly suggested that local environmental factors (particularly soil C:N ratio) contributed more in determining archaeal diversity than climate zone or continent^15^. In the McMurdo Dry Valleys, soil water content was as apparently a main driver for the archaeal community richness^19^. In a study of Chinese soils, based on canonical correspondence analysis, the distribution and diversity of archaeal communities was found to be primarily influenced by soil pH^20^. An investigation of elevational distribution patterns of soil archaeal communities in Mountain Shegyla in China found that 75.4% of the community variance could be explained by soil geochemical factors^21^. Tripathi *et al*.^22^ compared soil archaeal communities in moist climates in tropical and temperate eastern Asia, and found evidence that environment in terms of both climate and soil pH has a strong influence on archaeal community structure. These latter studies indicated that contemporary environmental factors, rather than dispersal lag and local evolution, are more important in shaping the soil archaeal community structure.

Although interesting, most of these studies (with the exception of 18) have focused on relatively outdated molecular technology that either gives only crude taxonomic resolution, or conversely a narrow taxonomic focus on particular taxa of archaea, or relatively small numbers of reads overall. For example, in the global comparison of soil archaea by Bates *et al*.^15^, only 2% of the sequences obtained by 454 sequencing were archaeal (the rest being bacteria), due to the generalized 16 S primer that was being used, and the much lower relative abundance of archaea compared to bacteria in soils. Lack of taxonomic breadth or precision, or low numbers of reads, in most of these previous studies are a serious impediment to understanding the broad scale patterns in archaeal communities, and the influences on community structure.

Here, we chose the eastern Tibetan Plateau as an area of investigation, partly because it has been very little studied from the point of view of soil archaea, and also because it presents a strong aridity gradient from east to west (less than 100 mm annual precipitation in the north west to greater than 800 mm in the south east, with an overall mean across the region of ~400 mm^23^). The Tibetan Plateau is the youngest (~2.4 × 10^8^ years), largest (~2.0 × 10^6^ km^2^) and highest (~4000 m on average) plateau in the world. Due to its extreme environmental conditions, microbes in these soils might be expected to harbor relatively distinctive microbial communities. In this study, we set out to investigate the following questions: 1) What are the dominant archaeal taxa in Tibetan soils? 2) How is the archaeal community distributed across the Tibetan Plateau soils? Can variation in archaeal community be explained in terms of environment alone, without invoking distance and dispersal history as a part of the explanation?

## Results

### Soil archaeal community composition

**Figure 1:**
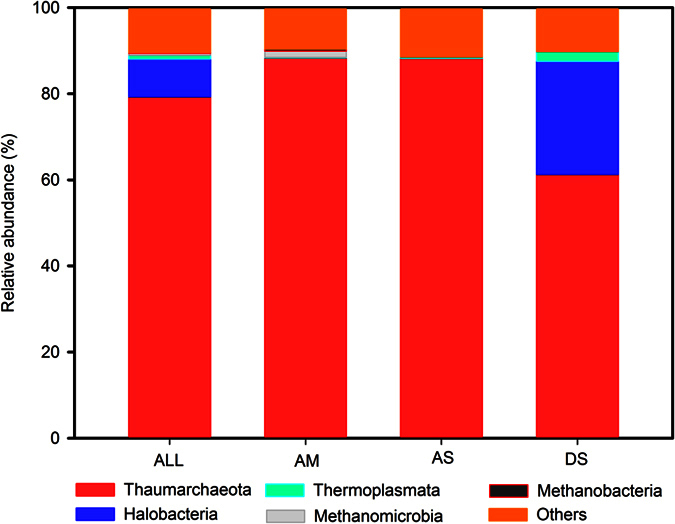
Relative abundance of the dominant archaeal phyla/genus in all soils combined, and in soils separated according to vegetation types categories. Abbreviations: All: all the soil samples; AM: Alpine Meadow; AS: Alpine Steppe; DS: Desert Steppe.

After denoising and chimera checking, we obtained 464,890 sequences that ranged from 1,363 to 22,166 per sample (mean = 4,947) and were able to classified 99.8% of these sequences. The dominant archaeal phyla were Thaumarchaeota (79.27% of sequences) and Halobacteria (8.75%), accounting for more than 88% of the archaeal sequences in each soil (Fig. 1, Table S1). Thaumarchaeota was relatively least abundant (61.26%) in desert steppe (DS), while Halobacteria was highest (26.24%) in this arid environment (Table S1). Methannomicrobia, Thermoplasmata, and Methanobacteria belonging to Euryarchaeota were also detectable at low levels of relative abundance.

### Influence of soil properties on soil archaeal communities

**Figure 2:**
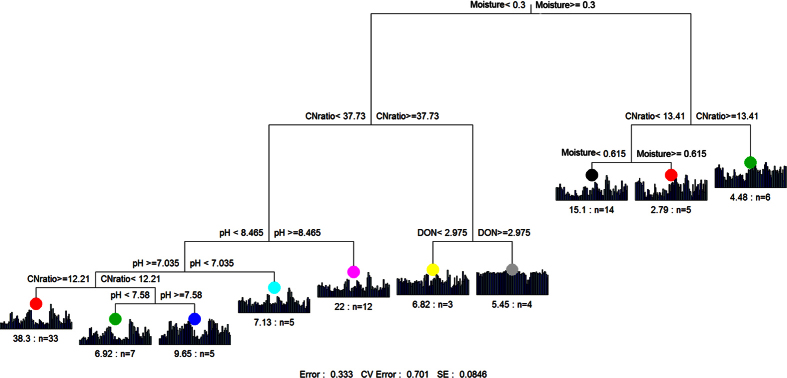
Multivariate Regression Trees (MRT) analysis of the archaeal community data associated the environmental variables.

**Figure 3:**
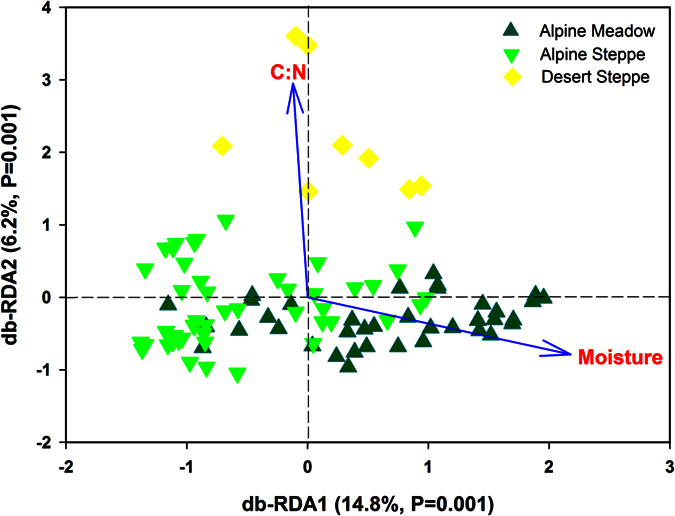
Ordination of soil archaeal community data, db-RDA using soil moisture and C:N ratio as environmental variables. Sites have been color coded according to vegetation type.

In terms of OTU composition (randomly selected 1300 sequences per sample), the archaeal community differed between the three main vegetation types: alpine steppe (AS), alpine meadow (AM), and desert steppe (DS) (Fig. S1) and this was confirmed by ANOSIM analysis, (Table S2). We found that the archaeal community structure was significantly correlated with soil characteristics (e.g. soil moisture, C:N ratio, inorganic C, total C, organic C, total N, and pH) (Table S3). Among the three vegetation types, soil C:N ratio was lower and moisture was higher in the AM than in the other two vegetation types (Fig. S2). To discern the relative importance of these soil characteristics in shaping soil archaeal community, multiple regression analysis (MRT) was used and the results showed that the soil archaeal community was strongly influenced by soil moisture and C:N ratio (Fig. 2). Using distance based RDA analysis, we confirmed that the composition of the soil archaeal community could be strongly influenced by the soil moisture (explanation of the variation: 14%, P = 0.001) and C:N (7%) (Fig. 3). Regardless of the community metric studied, the archaeal phylotype richness, measured as OTUs (> = 97% similarity), was negatively correlated with soil moisture(r = −0.47, p < 0.01) (Table 1). Other soil characteristics, such as total nitrogen, total carbon, soil organic carbon, dissolved total nitrogen, NH_4_^+^-N and NO_3_^−^-N were also negatively correlated with archaeal OTU richness (Table 1). Together, these results suggest that soil moisture could be a driving factor for soil archaeal community composition and phylotype richness across the eastern Tibetan Plateau.

### The relative influence of soil properties and spatial distance on soil archaeal communities

**Figure 4:**
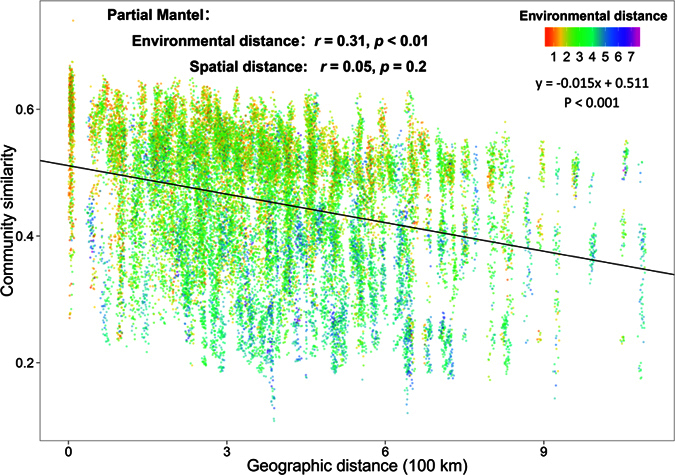
Distance-decay curves of similarity for the archaeal communities. Environmental distance were fitted on the archaeal community similarity. The relationships between archaeal community and environmental or spatial distance were evaluated by Partial Mantel test.

In order to compare the relative role of geographic distance and environmental distance on the community similarity, the distance-decay of archaeal communities was calculated, and environmental distance was compared with the archaeal community similarity. Also, the significance of the relationship between community dissimilarity and geographical distance vs environmental dissimilarity was assessed by a Partial Mantel test. We found a strong distance-decay relationship across our sampled area (Fig. 4), and also, the community similarity decreased with increasing environmental distance. However, we found the community similarity was overwhelmingly influenced only by the environmental factors (Fig. 4), because archaeal community composition showed no relationship with geographic distance according to the Partial Mantel test. This suggests that spatial distance alone is less important than local environmental factors in determining archaeal community differences in Tibetan Plateau soil environments.

## Discussion

We found that Thaumarchaeota was the dominant archael phylum in all three main vegetation types across the >900 km study area, while the relative abundance of Halobacteria was the highest in the desert steppe soils (Fig. 1).

Halobacteria abundance was recently reported to be mostly influenced by salinity:^24^ members of this phylum are found in high-pH soda lakes^25^, Mg^2+^-rich water bodies^26^, solar salterns^3^ and crystallizer ponds^27^. In the present study, the salinity, high pH conditions and strong sun exposure could be a large part of the reason why Halobacteria were more prevalent in the arid environments^7^. The dominance of Thaumarchaeota in all our samples (Fig. 1) is true to patterns observed in soils elsewhere^28^. Thaumarcheota are a mesophilic group, now recognized as a third archaeal phylum in 2008^29^,^30^. It is generally supposed that most soil Thaumarchaea have ammonia oxidizing ability and collectively play a significant role in nitrogen cycling^31^,^32^. Many studies have found that Thaumarchaeota are the most common group of archaea in terrestrial and aquatic habitats^29^,^33^,^34^. This suggests that at least a the broadest taxonomic level, the semi-arid and high-elevation Tibetan Plateau environments tend to harbor soil archaeal communities that are relatively common in other land habitats around the world.

In our study, the archaeal community differed between the three main vegetation types (Alpine Meadow (AM), Alpine Steppe (AS) and Desert Steppe (DS)) (Fig. S1), whose distribution relative to one another is determined by precipitation and moisture availability. Although many studies have suggested that plant community structure can affect soil bacterial community variation^35^,^36^,^37^,^38^, for archaea this has been found only in the study by Angel *et al*.^18^ in Israel. Since all the available evidence suggests that soil archaea are neither plant symbionts nor strictly dependent on soil organic matter for energy^34^,^39^, is likely however that soil moisture availability - which also brings about the gradient in vegetation types - is more directly important in determining archaeal community structure, and that the relationship to plant community composition is very indirect or purely incidental.

Numerous studies have shown strong correlations between soil moisture and macroorganism community structure^40^, and it is not surprising to see this also in the microbial world. For example, Zhang *et al*.^41^ found that bacterial diversity (H’) significantly correlated with soil moisture in the Tibetan permafrost region. In a high Arctic polar oasis, Banerjee *et al*.^42^ found soil moisture was the key edaphic factor which drove the archaeal community structure. Angel *et al*.^18^ found strong gradients in both bacterial and archaeal community composition across the soil moisture gradient in Israel. Consistent with our study, archaeal diversity across this study region (standardized for number of reads) was also most closely related to soil moisture, with diversity being greatest in the driest areas^18^. A possible reason is that drought stress promotes some rare archaeal phyla, increasing their numbers to levels at which they can be detected in our study, and reflected in the increased diversity. Typically for studies of soil archaeal phylotype richness, our rarefaction curves for archaeal OTU richness (Fig. S3) did not reach an asymptote, even at an average of 4,947 quality sequences per sample. This indicates that an unknown number of rare archaeal OTUs are missed by this survey, even though the major components of the community can be compared with confidence. Additionally beyond moisture conditions, soil C:N ratio also showed a strong influence on the soil archaeal community composition in the Tibetan soils. The importance of C:N ratio for bacterial community has been well documented in other studies elsewhere^4^,^5^,^6^,^41^. In a recent study, C:N ratio was found to be the best predictor for both surface and subsurface bacterial community distribution in western Tibetan Plateau soils^7^. This might also be expected for soil Archaea, since soil C:N ratio was the only factor consistently correlated with archaeal community structure and diversity in a global scale study of soil archaeal communities^15^. However, our MRT (Fig. 2) and db-RDA (Fig. 3) analyses clearly demonstrated soil moisture as the best predictor for variation in archaeal community composition across the Tibetan Plateau, and soil C:N ratio may be of intermediate importance after moisture in determining archaeal communities.

The fact that the community distributions of archaea across our sample area can be explained in terms of identifiable environmental factors, suggests that despite the long history of dramatic environmental changes in the Tibetan Plateau^43^, it is not necessary to hypothesize any dominant role for dispersal rates and the vagaries of history in determining variation in soil archaeal community structure (Fig. 4). It appears that in the case of Archaea – at least in our Tibet study area -‘everything is everywhere, but the environment selects’^8^. The predominant influence of present-day environmental variables (rather than dispersal history or recent evolutionary history) in determining broad scale community variation in soil microbes is also evident in various studies of bacterial communities in American^4^, Arctic^5^ and British soils^6^. In the case of bacteria, in these other studies, the residual spatial variation unexplained by measured environmental factors tends to be greater, leaving greater room for a dispersal limitation effect. However, this unexplained variation could merely reflect the effects of unknown environmental factors that vary spatially. In the present study of archaea, the relatively strong explanatory power of a few measured environmental variables in predicting community composition might perhaps reflect the unimportance of many soil and biotic factors in affecting archaeal ecology, and the dominant influence of just a few key factors^34^.

## Conclusion

Understanding the controls on archaeal community structure may be significant in predicting the effects which archaea can potentially have in providing labile nitrogen for developing ecosystems as climate warming occurs.

Recently, widespread and rapid degradation of permafrost has been occurring due to climate warming, and these changes may significantly alter soil moisture content and soil nutrient availability^44^, and may possibly release of massive amounts of carbon into the atmosphere^41^. The substantial soil carbon reservoir on the Tibetan Plateau^45^ may become labile due to thawing permafrost and accelerated microbial metabolism^46^,^47^. The indications of distinct community structures of archaea suggest a fine degree of adjustment to certain key environmental factors, in that different combinations of OTUs thrive in different sets of soil moisture conditions. If conditions change, new combinations of archaeal OTUs may be necessary for nitrogen cycling to operate most effectively. However, the lack of any major dispersal lag, as indicated by the spatial analyses performed here, suggests that archaea can adjust their distributions quite rapidly - at least on the time scale of centuries and possibly on shorter timescales - when the environment changes^48^,^49^,^50^, providing some reassurance on the responsiveness and resilience of these high altitude ecosystems

Lack of evidence for dispersal lag in the Tibetan Pleateau region suggests that adjustment of functional communities of archaea has been possible at least on the time scale of centuries-to-millennia on which past climate changes have occurred^51^.

## Method and Materials

### Sample collection, DNA extraction, and soil characterization

94 soil samples were collected from 36 sites, representing three main vegetation types (Alpine steppe, Alpine meadow, Desert steppe) in 2011 (Fig. S4). All samples were collected during the peak growing season, from natural soils that were minimally disturbed. At most sites, we sampled three plots 40 meters apart, and in all samples we collected 5–7 cores per plot at a depth of 0–5 cm, which were subsequently combined. For a total of 94 composite soil samples, soil DNA was from 0.5 g soil, using the Power Soil kit (MO BIO laboratories, Carlsbad, CA) according the manufacture’s instruction and storing at −40 °C. The extracted DNA was diluted to nearly 25 ng/μl with distilled water and stored at −20 °C until PCR. 2 μl of diluted DNA sample of each plot were used as template for amplification; the V3–V5 hyper variable regions of archaeal 16SrRNA were amplified using the primer set: Arch344F: 5′-ACGGGGYGCAGCAGGCGCGA-3′ with the Roche 454 ‘A’ pyrosequencing adapter and a unique 7 bp bar-code sequence, and primer Arch915R: 5′-GTGCTCCCCCGCCAATTCCT-3′ with the Roche 454 ‘B’ sequencing adapter at the 5′-end of each primer respectively. Each sample was amplified in triplicate with 50ul reaction under the following conditions: 94 °C for 5 min, 10 cycles of touchdown PCR were performed (denaturation at 94 °C for 30 s, annealing for 30 s with an 0.5 °C/cycle decrement at 61 °C above the respective annealing temperatures and extension at 72 °C for 1 min), followed by 25 cycles of regular PCR (94 °C for 30 s, 30 s at the respective annealing temperature, and 72 °C for 1 min and a final extension step for 7 min at 72 °C^52^. PCR products from each sample were pooled together and purified by Agarose Gel DNA purification kit (TaKaRa) and then combined in equimolar ratio in a single tube and run on a Roche FLX454 pyrosequencing machine (Roche Diagnostics Corporation, Branford, CT, USA), producing reads from the forward direction Arch344F. All of the soil variables and location information were described in Table S4.

### Pyrosequencing data analyses

Data were processed and analyzed following the procedure described in Hamady *et al*.^53^ and Chu *et al*.^5^ using Quantitative Insights Into Microbial Ecology (QIIME) pipeline (http://qiime.sourceforge.net/)^54^. All the remaining raw data were denoised using two low level scripts from QIIME^55^. Operational Taxonomic Unit (OTU) picking, filtering, chimera checking, and clustering (based on 97% similarity) were performed with QIIME using USEARCH^56^,^57^. Specifically, chimeric sequences were removed using a combination of de novo and reference-based chimera checking with the flags –non_chimeras_rentention = intersection. A representative sequence was chosen from each phylotype by selecting the most highly connected sequence^53^. All representative sequences were aligned by PyNAST^58^. Taxonomic identity of each phylotype was determined using the Greengenes database (http://greengenes.lbl.gov/).

### Statistical analyses

We calculated the richness (i.e. number) of phylotypes from each sample to compare the community level diversity at a single level of taxonomic resolution. Correlations between phylotype richness and soil characters were conducted by SPSS 20.0 for windows. Using the soil archaeal community data (OTUs-Table 1,300 sequences randomly selected), Nonmetric multidimensional scaling (NMDS) ordinations were generated using *monoMDS ()* function in the vegan tool of R version 2.3.0^59^ on the basis of Bray-Curtis dissimilarities. In addition, significant differences in community composition among the vegetation types were tested using analysis of similarities (ANOSIM) with R^59^. Based on the OTUs-Table_1300, Multivariate Regression Trees (MRT) plot was used to show community-environment relationship constrained by the key environmental variables using “mvpart” package in R 2.3.0^59^. Before MRT analysis, autocorrelations among soil factors were considered and variables with VIF (variance inflation factor) <20 were selected using *vif ()* function in R 2.3.0^59^. Then the most influential factors which were included in the MRT analysis were selected by *bioenv ()* function in R 2.3.0^59^. Distance-based redundancy analysis (db-RDA) was performed using “capscale ()” function in Vegan packages of R 2.3.0^59^ based on dissimilarity calculated using the Bray-Curtis index, and soil moisture (pseudo-F = 16.1, P = 0.001, Number of permutations: 999) and C:N ratio (pseudo-F = 8.1, P = 0.001, Number of permutations: 999) were selected as a best solution for the db-RDA ordination^60^ 0. The Mantel test was performed to find the relationship between the soil archaeal community and each soil factor, and the distance matrix were Bray Curtis distance of soil archaeal community data (OTUs-Table 1,300 sequences randomly selected) and Euclidean distance of each environmental variable. Partial Mantel Test was used to explain the correlation between archaeal community composition and the soil factors and spatial factors^61^. Distance decay curve was calculated according to Nekola and White^62^.

### Data availability

Sequences generated in this study have been deposited in the European Molecular Biology Laboratory (EMBL) under accession number ERP009034 (http://www.ebi.ac.uk/ena/data/view/ERP009034).

## Additional Information

**How to cite this article**: Shi, Y. *et al*. The biogeography of soil archaeal communities on the eastern Tibetan Plateau. *Sci. Rep.* **6**, 38893; doi: 10.1038/srep38893 (2016).

**Publisher's note:** Springer Nature remains neutral with regard to jurisdictional claims in published maps and institutional affiliations.

## Supplementary Material

Supplementary Information

## Figures and Tables

**Table 1:** Correlations (*r*) between phylotype richness of archaea and the characteristics of soil and plant. Values in bold are statistically significance at *p* < 0.05.

	*r*	*p* value
SM	**−0.47**	<0.01
SOC	**−0.36**	<0.01
TN	**−0.36**	<0.01
pH	−0.08	0.46
TC	**−0.36**	<0.01
SIC	0.06	0.56
DTN	**−0.24**	0.02
NH_4_^+^-N	**−0.28**	0.01
C:N ratio	0.1	0.34
DOC	−0.09	0.39
DON	−0.14	0.17
NO_3_^−^N	**−0.25**	0.02
Plant species richness	−0.1	0.32
Plant Shannon index	−0.06	0.57

Abbreviations: SM: soil moisture content; SOC: soil organic carbon content; TN: total nitrogen content; TC: total carbon content; SIC: soil inorganic carbon; DTN: dissolve total nitrogen; C:N ratio: soil carbon and nitrogen ratio; DOC: dissolved organic carbon; DON: dissolved organic nitrogen.
