# Supplementary material for: The biogeography of soil archaeal communities on the eastern Tibetan Plateau
Source: Sci Rep. 2016 Dec 13;6:38893. doi: 10.1038/srep38893 (PMC5153633; doi:10.1038/srep38893)

**Title Page**

The biogeography of soil archaeal communities on the eastern Tibetan Plateau

Yu Shi^1^, Jonathan M. Adams^2^, Yingying Ni^1^, Teng Yang^1^, Xin Jing ^3^, Litong Chen^4^, Jin-Sheng He ^3,4^ & Haiyan Chu^1^*

^1^State Key Laboratory of Soil and Sustainable Agriculture, Institute of Soil Science, Chinese Academy of Sciences, East Beijing Road 71, Nanjing 210008, China

^2^Department of Biological Sciences, Seoul National University, Gwanak, Seoul 151, Republic of Korea

^3^Department of Ecology, College of Urban and Environmental Sciences and Key Laboratory for Earth Surface Processes of the Ministry of Education, Peking University, 5 Yiheyuan Road, Beijing 100871, China

^4^ Key Laboratory of Adaptation and Evolution of Plateau Biota, Northwest Institute of Plateau Biology, Chinese Academy of Sciences, 23 Xinning Road, Xining 810008, China

***Corresponding author:** Haiyan Chu Tel.: +862586881356, Fax: +8602586881000, E-mail: [hychu@issas.ac.cn](mailto:hychu@issas.ac.cn).

**Table S1**.Means of the relative abundances of phyla classified using the Greengenes database across all soils and soils grouped into three vegetation types (values represent % of total non-redundant sequences).

|  | All | AM | AS | DS |
| --- | --- | --- | --- | --- |
| **Thaumarchaeota** | **79.27** | **88.32** | **88.21** | **61.26** |
| **Euryarchaeota** | **10.29** | **2.03** | **0.32** | **28.5** |
| Halobacteria | 8.75 | 0 | 0 | 26.24 |
| Thermoplasmata | 0.9 | 0.13 | 0.31 | 2.25 |
| Methanomicrobia | 0.5 | 1.49 | 0.01 | 0 |
| Methanobacteria | 0.14 | 0.41 | 0 | 0 |
| **Others** | **10.45** | **9.65** | **11.47** | **10.23** |

Abbreviations: All: all the soil samples; AM: Alpine Meadow; AS: Alpine Steppe; DS: Desert Steppe.

Table S2. Dissimilarities in archaeal community composition between vegetation types on Tibetan Plateau as determined by ANOSIM analysis. For the abbreviations, please see Table S1.

|  | R | P |
| --- | --- | --- |
| AM vs. AS | 0.33 | 0.001 |
| AM vs. DS | 0.66 | 0.001 |
| AS vs. DS | 0.74 | 0.001 |

**Table S3**.Relationship between archaeal community and soil characteristics derived from mantel test.

|  | *r* | *p* |
| --- | --- | --- |
| C:N ratio | **0.31** | <0.01 |
| SM | **0.27** | <0.01 |
| SIC | **0.20** | <0.01 |
| TC | **0.15** | 0.02 |
| SOC | **0.15** | 0.01 |
| TN | **0.14** | 0.01 |
| pH | **0.09** | 0.02 |
| NO_3_^-^-N | 0.07 | 0.14 |
| DOC | 0.06 | 0.17 |
| DTN | 0.05 | 0.2 |
| NH_4_^+^-N | 0.03 | 0.27 |
| DON | 0 | 0.6 |

Abbreviations: SM: soil moisture content; SOC: soil organic carbon content; TN: total nitrogen content; TC: total carbon content; SIC: soil inorganic carbon; DTN: dissolve total nitrogen; C:N ratio: soil carbon and nitrogen ration; DOC: dissolved organic carbon; DON: dissolved organic nitrogen.

**Table S4** Description of the geographic and soil variables according to the Vegetation types, the values are minimum-maximum ranges. The abbreviations are shown in Table S3.

|  |  | Vegetation type |  |
| --- | --- | --- | --- |
|  | Desert steppe | Alpine meadow | Alpine steppe |
| Longitude | 94.5-97.1 | 91.1-100.2 | 91.1-101 |
| Latitude | 35.9-37.5 | 30.5-35.7 | 31.5-37.3 |
| Altitude | 2918-3633 | 3728-4702 | 2938-4608 |
| Soil moisture | 0-0.07 | 0.01-1.54 | 0-0.15 |
| pH | 7.83-8.88 | 6.59-8.79 | 7.14-9.15 |
| TC | 1.55-2.92 | 1.73-15.96 | 0.72-4.89 |
| SIC | 0.81-1.55 | 0-3.18 | 0.01-1.19 |
| SOC | 1.45-2.73 | 1.72-15.95 | 0.71-4.87 |
| TN | 0.02-0.07 | 0.07-1.19 | 0.03-0.44 |
| C:N ratio | 32.88-75 | 10.51-32.29 | 9.09-36.38 |
| DOC | 98.15-327.28 | 0.75-838.12 | 29.46-425.65 |
| DTN | 9.36-45.5 | 10.27-134.85 | 6.5-62.13 |
| DON | 0.61-14.12 | 0.95-54.9 | 0-34.36 |
| NO_3_^-^-N | 0.86-29 | 0-84.13 | 0-36.26 |
| NH_4_^+^-N | 5.48-9.19 | 5.23-29.14 | 4.56-16.19 |
| Sample number | 8 | 38 | 48 |

**Figure S1**. Archaeal community structure in the Tibetan Plateau soils as indicated by non-metric multidimensional scaling plots (NMDS). Sites have been color coded according to vegetation type.


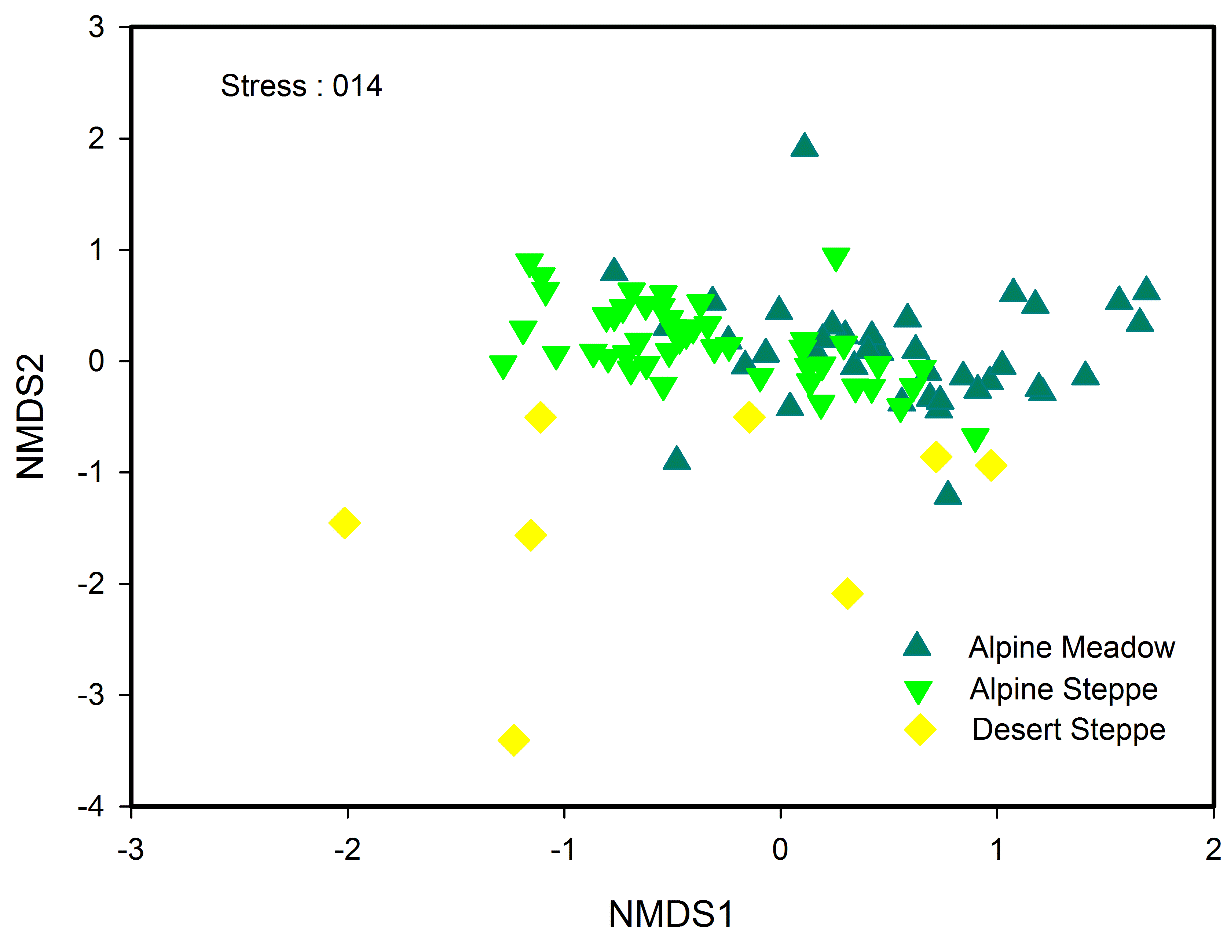


**Figure S2** Soil C:N ratio (left) and moisture (right) in Tibetan Plateau were compared by different vegetation types using Wilcoxon signed-rank test . Vertical bars are standard deviations. Different letters above bars indicate the values are significantly different among the vegetation types.


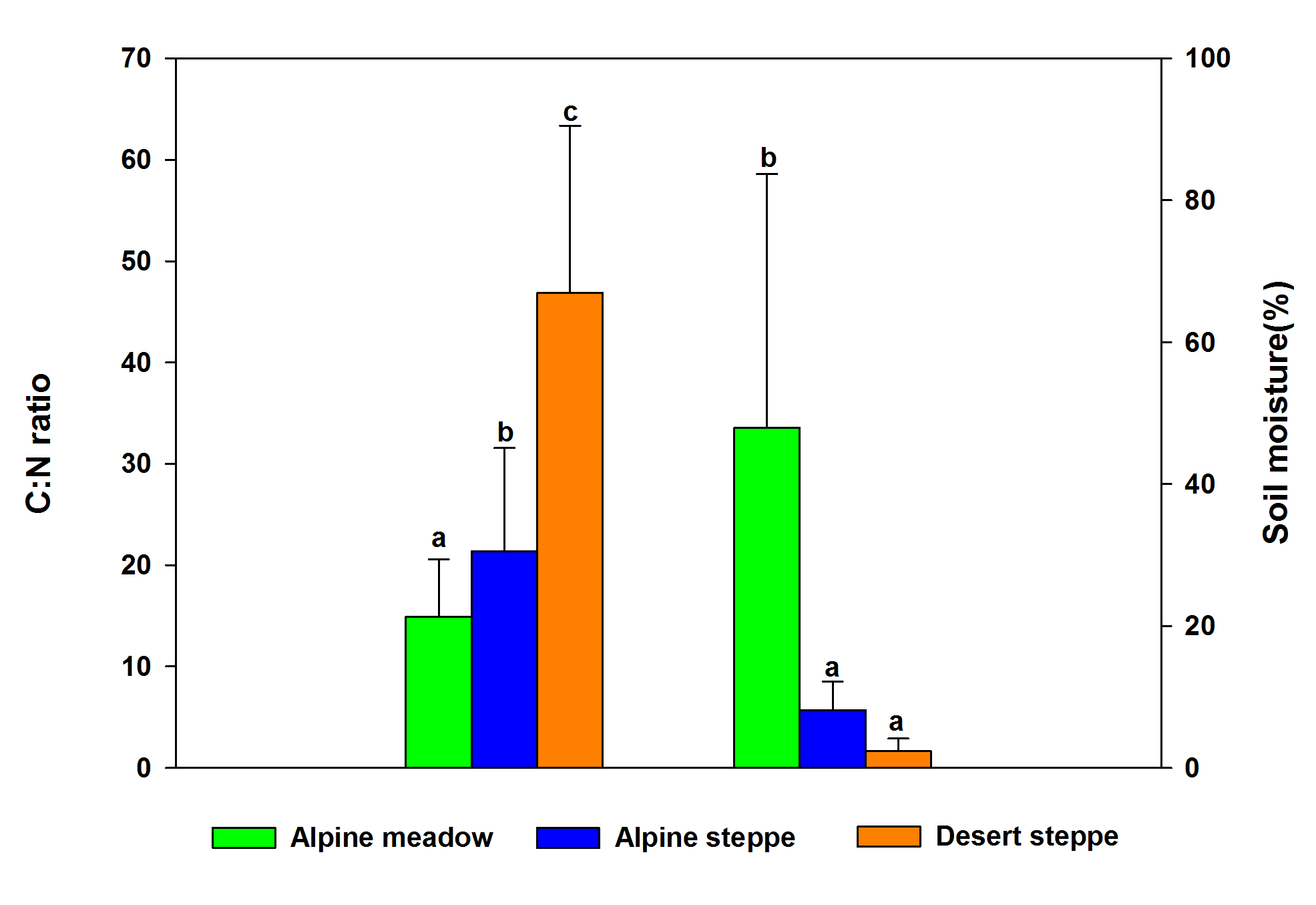


**Figure S3** Rarefaction curves of the phylogenetic diversity (A) and observed OTU richness (B).

**
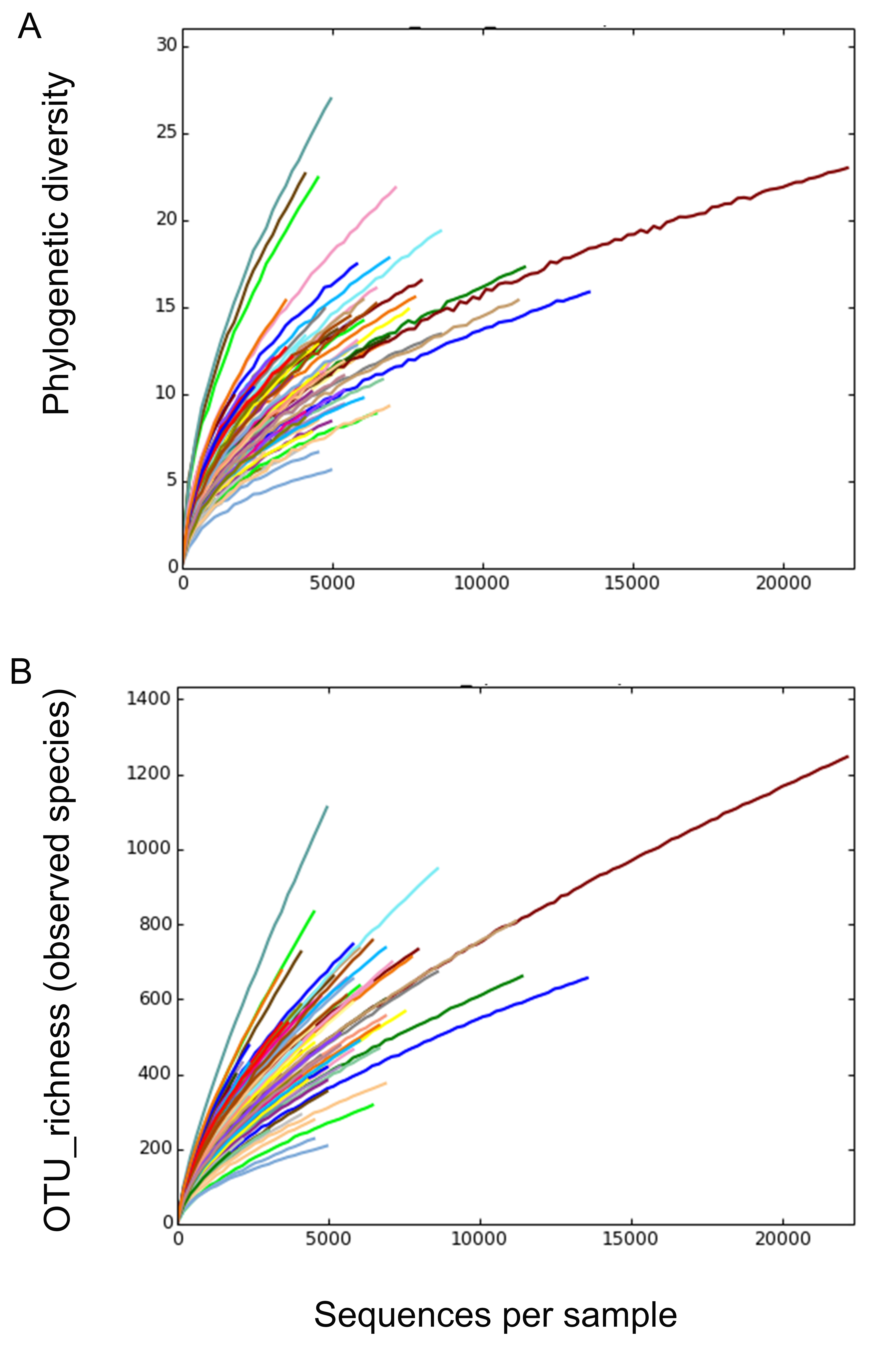
**

**Figure S4**. Locations of sampling sites on Tibetan Plateau. The map in was generated by 'map' function from the map database of China [Extra Map Databases. mapdata: R package version 2.2-6, <https://CRAN.R-project.org/package=mapdata>. Code: map ('china', col = "black", ylim = c(26, 40), xlim = c(78, 103), panel.first =grid(lwd=1.8)). Original S code by Richard A. Becker and Allan R. Wilks. R version by Ray Brownrigg. (2016).] in R.


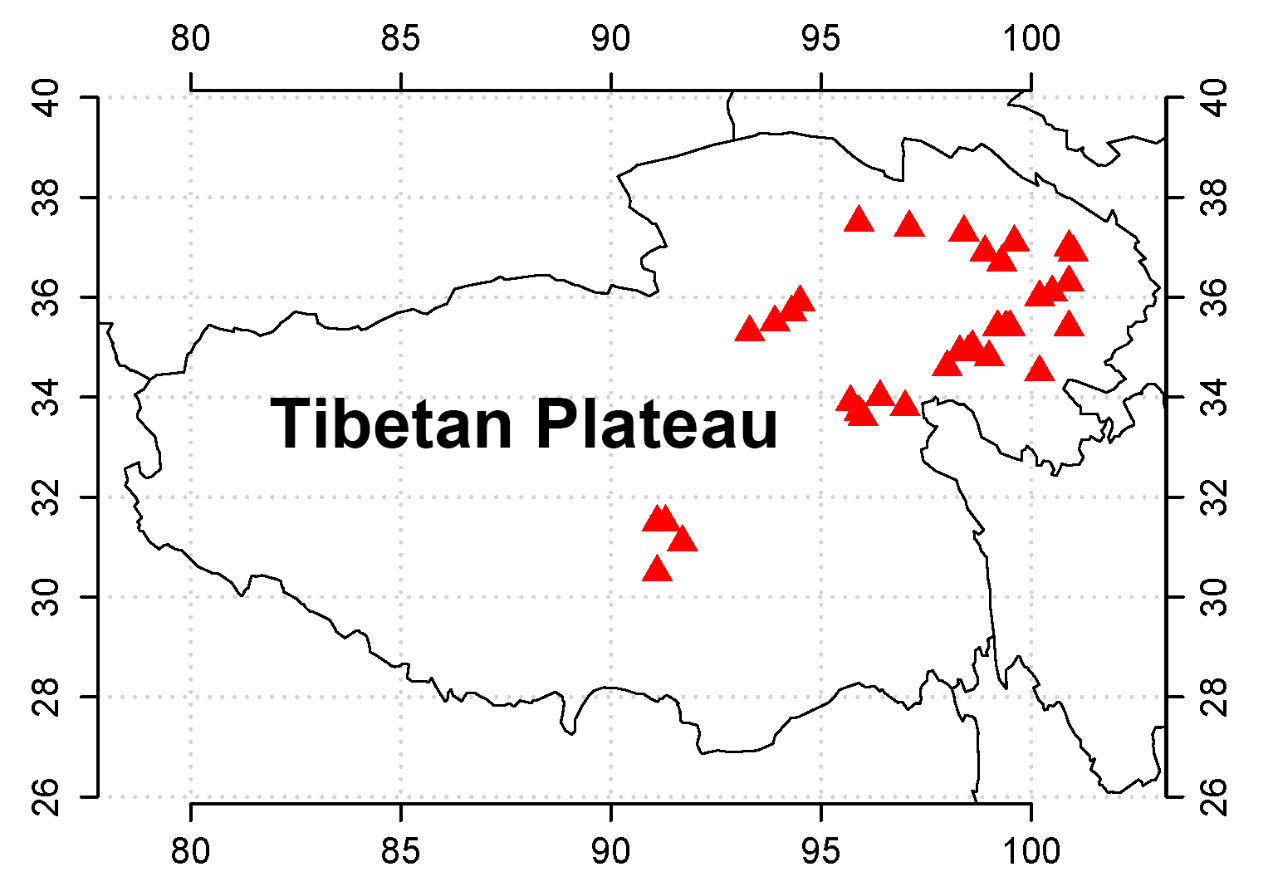

Supplement: Supplementary Information [file srep38893-s1.doc]
